# Clinical Neuroscience Research in Saudi Arabia: A Comparative Evaluation of Performance at Country and Worldwide Levels Based on the Relative Specialization Index

**DOI:** 10.7759/cureus.68842

**Published:** 2024-09-06

**Authors:** Abdulhakim B Jamjoom, Abdulhadi Y Gahtani, Jude M Jamjoom, Belal M Sharab, Yousuf K Khogeer, Ohood H Alshareef, Moajeb T Alzahrani

**Affiliations:** 1 Department of Neurosurgery, King Saud Bin Abdulaziz University for Health Sciences College of Medicine, Jeddah, SAU; 2 College of Medicine, Alfaisal University, Riyadh, SAU; 3 College of Medicine, Yildirim Beyazit University, Ankara, TUR; 4 College of Medicine, University of Central Lancashire, Preston, GBR; 5 Department of Pharmaceutical Care Services, King Abdulaziz Medical City Western Region, Jeddah, SAU

**Keywords:** bibliometrics, clinical neuroscience, medical specialty research, relative specialization index, saudi arabia, scimago journal and country rank, worldwide ranking

## Abstract

This review is an appraisal of the performance of clinical neuroscience research in Saudi Arabia based on the measurement of the Relative Specialization Index (RSI). The latter is an established quantitative performance indicator that determines whether a country has a relatively higher or lower share in world publications in a specialty than its overall part in the world total publications. The study aimed to assess the trends in the specialty’s RSI, comparing it to other medical specialties in Saudi Arabia and to that of the top 50 countries worldwide in clinical neuroscience. SCImago Journal and Country Rank were used to determine the total articles and total citations for 46 medical specialties in Saudi Arabia and clinical neuroscience in the worldwide top 50 countries during 1996‑ 2023. The RSI was calculated for each medical specialty and each country. A positive or negative RSI implied that the specialty’s share in the country’s total documents or total citations was higher or lower than the average for the specialty worldwide. A steady increase in Saudi Arabia’s total articles and total citations in clinical neuroscience was observed over the last 28 years. The RSI values, however, remained negative throughout except for limited periods (2003-2006 for total articles) and (1996 and 1998 for total citations). Compared to other medical specialties in Saudi Arabia, the specialization performance for clinical neuroscience was within the mid-range in total articles (ranking 30^th^ out of 46 specialties) and the low range in total citations (ranking 39^th^ out of 46 specialties). Saudi Arabia’s worldwide ranking in clinical neuroscience based on total citations was 39; however, the country’s ranking dropped to 45 when the RSI values were applied. Furthermore, clinical neuroscience was considered to have had a strong relative contribution (RSI ≥ 0.1) to the total articles in five countries (Italy, Austria, Germany, Japan, and Canada) and total citations in six countries (Luxembourg, Austria, Germany, Canada, Italy, and Finland). In conclusion, despite an increase in Saudi Arabia’s total articles and total citations in clinical neuroscience over the years, the specialty’s relative share of the total productivity in the country remains lower than the overall for the specialty worldwide. The performance of the specialty was within the mid-to-low range compared to the other 45 medical specialties in Saudi Arabia. In addition, the country's worldwide ranking based on total citations in the specialty dropped when the RSI was used. Clinical neuroscience researchers in Saudi Arabia are encouraged to improve the quality and quantity of their research productivity to be one of the leading medical specialties in Saudi Arabia.

## Introduction and background

Evaluation of medical specialties' global productivity and worldwide ranking have been the subject of several publications and remain a matter of interest [1-5]. SCImago Journal and Country Rank (SJR) [6] is an online free-access portal that utilizes the Scopus database and provides lists of worldwide rankings for countries and journals based on several bibliometric indicators [1-3,7]. The Relative Specialization Index (RSI) is an established quantitative performance indicator that determines whether a country has a relatively higher or lower share in world publications in a specialty than its overall part in the world total publications. It is derived from the Activity Index (AI), which was first introduced by Frame in 1977 and further developed by others [8,9]. The index allows for benchmarking the position of a country in a specific specialty against the world’s average. Specialties in a country where RSI>0 indicate relative specialization in that particular field. The overall RSI score for a country should always be 0, which means that positive RSI values must always be balanced by negative ones [8,9]. In recent years, few studies reported the use of RSI measurements in assessing the specialization distribution in Saudi Arabia [2], the Netherlands, and China [8] and relating to tissue engineering in otolaryngology in several countries [10].

Over the last four decades, researchers from Saudi Arabia have contributed to the national and international medical and biomedical literature. Meo et al. [11]. reported that the country’s research performance in global medical sciences had markedly increased during the period 2006-2012, but the number of citations had decreased. Latif [12] described a linear progression in Saudi Arabia’s biomedical research production during 2008-2012, but most publications were in low Impact Factor (IF) journals. Al-Bishri [13] reviewed Saudi Arabia’s total publications during 2010-2011 and reported that most of the articles were in the fields of community medicine, pathology, medicine, and surgery. More recently, Ul Haq et al. [14] demonstrated that the total publications increased considerably from 1,332 in 2008 to 5,529 in 2017 and that medicine was the most prominent subject area. Furthermore, Vennu et al. [15] stated that the number of publications carried out by Saudi Arabia’s universities increased significantly from 73 in 2008 to 721 in 2017. However, most articles (80%) were published in journals with IF<3. The appraisal of Saudi Arabia’s research in clinical neuroscience has been limited to a few articles that analyze the country’s research production in epilepsy, neurology, and neurosurgery [16-18]. Three articles addressed Saudi Arabia’s productivity and worldwide ranking in clinical neuroscience [4,19,20]. However, the data reported in these articles were limited to 2018, and the specialty’s RSI was not measured. The purpose of the study was to evaluate clinical neuroscience’s share of total productivity in Saudi Arabia as measured by the RSI. The review aimed to highlight the changing trends in the specialty’s RSI over the years and compare it to other medical fields in Saudi Arabia and the top 50 countries in the specialty worldwide. The study also intended to identify the countries in which clinical neuroscience’s contribution to their total research was higher than the overall for the specialty worldwide.

## Review

Methods

This study was a review based on routinely available data with open access; hence, it did not require ethical approval by our institution. The SJR [6] was searched during July 2024 using the parameters "medicine," "clinical neurology," “each year from 1996 to 2023," and "all regions." The data collected was Saudi Arabia’s total articles and total citations, as well as the country’s worldwide ranking based on total articles and total citations in the specialty each year. A second SJR search was carried out utilizing the parameters "medicine," “each of the listed 48 medical specialties in the SJR website," “1996-2023," and “all regions." The subject categories ‘Drug Guides’ and ‘Medical Reviews and References’ were excluded due to limited participation by researchers from Saudi Arabia. The data collected was Saudi Arabia’s total articles and total citations, as well as the country’s worldwide ranking in each of the 46 medical specialties based on total articles and total citations during the searched period. A third search of the SJR website was performed employing the parameters "medicine," “clinical neurology," “1996-2023," and “all regions." The data collected were total articles worldwide and total citations for each of the top 50 countries in the world in the specialty during the searched period.

The subject category “clinical neurology” on the SJR website included 396 international journals that cover a range of clinical neuroscience specialties [6]. Hence, it was considered appropriate to refer to “clinical neurology” in the SJR site as “clinical neuroscience” thereafter in this article. The data obtained were used to calculate the AI and RSI based on total articles and total citations for clinical neuroscience in Saudi Arabia in each year from 1996 to 2023, for each of the 46 medical specialties in Saudi Arabia during 1996-2023, and for each of the top 50 countries worldwide in clinical neuroscience during 1996-2023. The AI and RSI were calculated using the following bibliometric formulae [2, 8, 10]:

AI = [(Specialty total articles/cites in Saudi Arabia) ÷ (All specialties total articles/citations in Saudi Arabia)] 

÷ 

[(Specialty total articles/citations in the world) ÷ (All specialties total articles/cites in the world)]

RSI = (AI-1) ÷ (AI+1)

The RSI has values that range from -1 to +1. An RSI>0 indicates above-world average productivity in the specialty, while an RSI<0 indicates below-world average. In this study, RSI≥0.1 in total articles or total citations was labeled as a strong relative contribution by the country to the specialty, whether clinical neuroscience or any other specialty. The RSI values of total articles and total citations for the top 50 countries were correlated with their worldwide rankings by calculating Pearson's correlation coefficient (R) using Social Sciences Statistics [21], with significance being reached when P<0.05.

Results

Data relating to the performance of clinical neuroscience in Saudi Arabia were analyzed in the three following categories.

Trends of the Years

Total articles and total citation rankings worldwide for clinical neuroscience in Saudi Arabia from 1996 to 2023 are shown in Table 1.

**Table 1:** Annual worldwide ranking for clinical neuroscience in Saudi Arabia based on total articles and total citations between 1996 to 2023

Year	Worldwide ranking (total articles)	Worldwide ranking (total citations)
1996	32	33
1997	37	37
1998	34	34
1999	36	38
2000	39	42
2001	45	44
2002	38	40
2003	35	39
2004	36	43
2005	38	47
2006	37	47
2007	41	52
2008	38	45
2009	41	52
2010	40	45
2011	40	39
2012	44	39
2013	39	41
2014	38	35
2015	39	40
2016	36	38
2017	35	38
2018	34	38
2019	34	34
2020	32	36
2021	34	35
2022	33	39
2023	28	37

**Figure 1:**
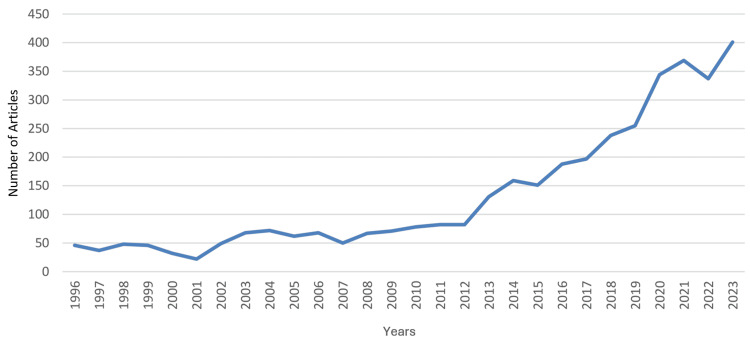
Saudi Arabia’s annual number of articles on clinical neuroscience between 1996 and 2023 This graph has been created by the authors.

**Figure 2:**
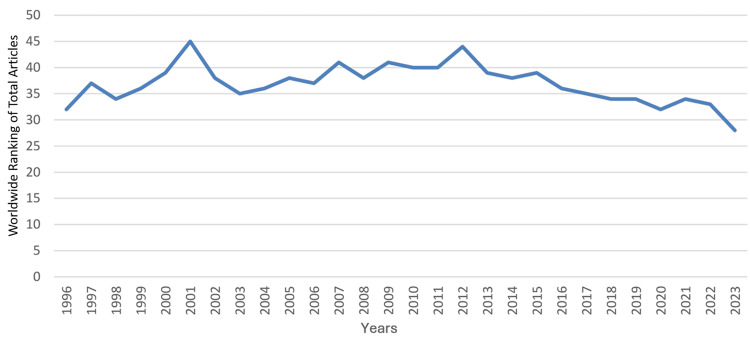
Worldwide ranking of Saudi Arabia’s annual total articles on clinical neuroscience from 1996 to 2023 This graph has been created by the authors.

**Figure 3:**
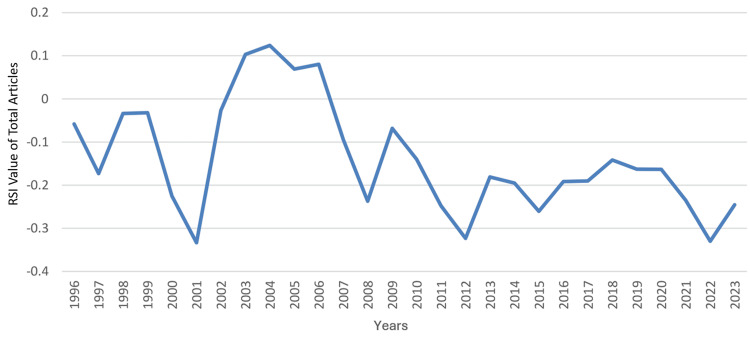
The RSI value of Saudi Arabia’s annual total articles on clinical neuroscience from 1996 to 2023 RSI: Relative Specialization Index This graph has been created by the authors.

During that period, the median (range) total articles and total citations were 75 (22 - 401) and 1,148 (275 - 7,403), respectively; the median (range) total articles and total citations worldwide rankings were 37 (28 - 45) and 39 (33 - 52), respectively; and the median (range) RSI values of total articles and total citations were -0.1681 (-0.3333 - 0.1241) and -0.2697 (-0.5671 - 0.031), respectively. A steady increase was observed in the country’s total articles that was most marked from 2012 to 2023. During that period, the annual number of articles increased from 82 to 401, which coincided with an improvement in Saudi Arabia’s worldwide ranking in the specialty from 44 to 28. However, this did not correspond with an increase in the RSI value of total articles, which remained negative throughout the period, ranging from -0.3234 to -0.2454. The RSI value of total articles was noted to be positive for a relatively short period (2003-2006). The trends in the performance of clinical neuroscience in Saudi Arabia are illustrated in Figure 1 for total articles, Figure 2 for worldwide rankings, and Figure 3 for RSI values.

**Figure 4:**
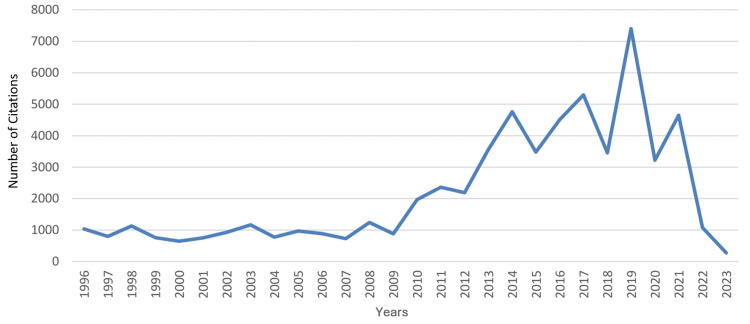
Saudi Arabia’s annual number of citations in clinical neuroscience during the years 1996-2023 This graph has been created by the authors.

**Figure 5:**
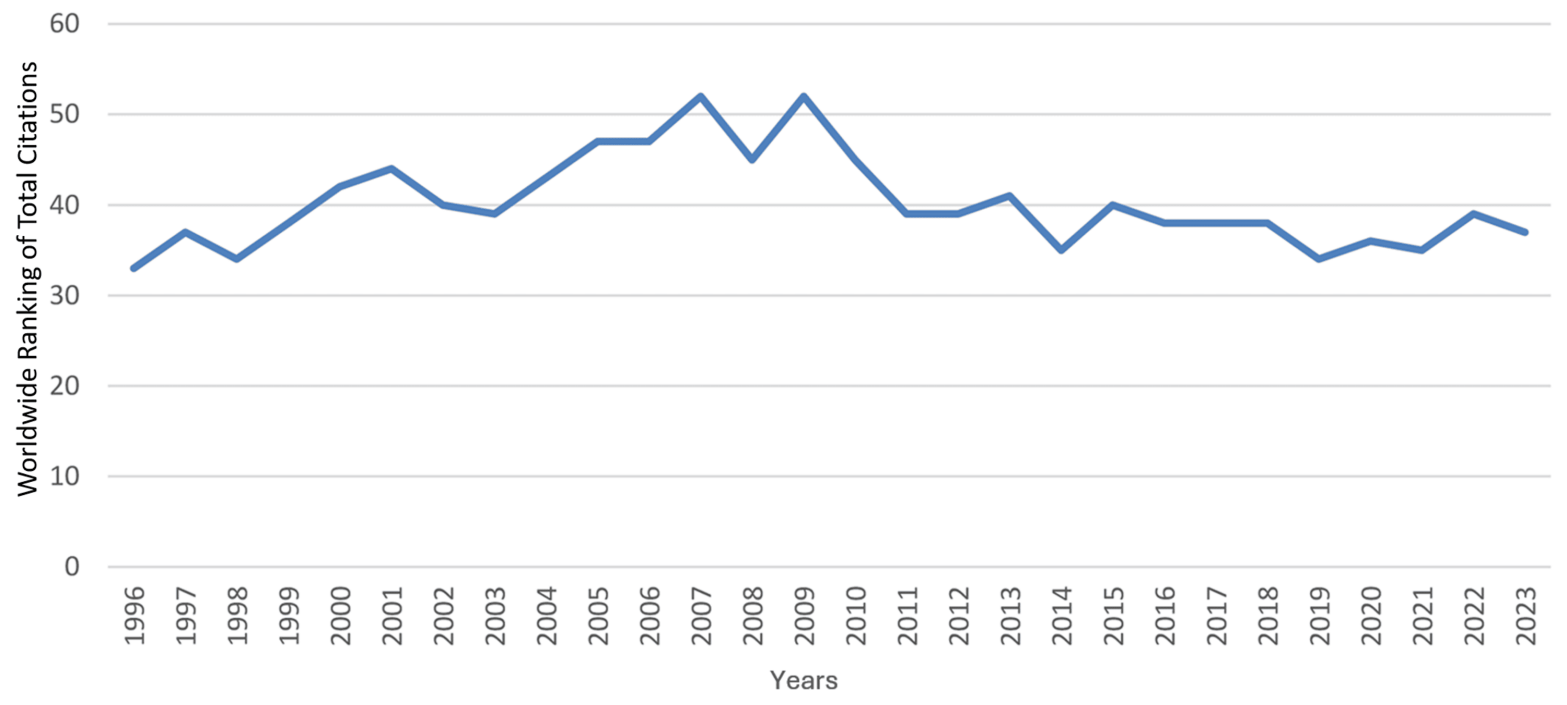
Worldwide ranking of Saudi Arabia’s annual total citations in clinical neuroscience from 1996 to 2023 This graph has been created by the authors.

**Figure 6:**
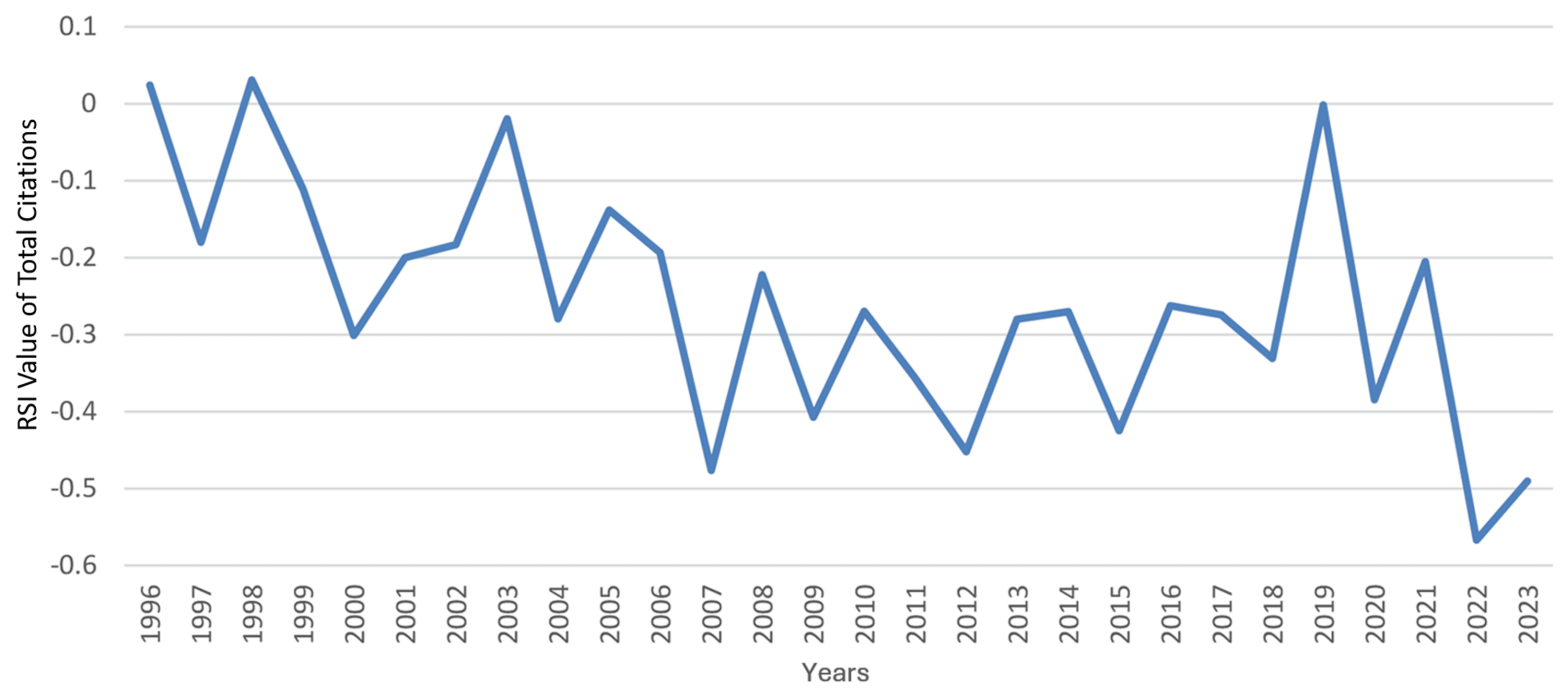
The RSI values of Saudi Arabia’s annual total citations in clinical neuroscience between 1996 and 2023 RSI: Relative Specialization Index This graph has been created by the authors.

An obvious increase was noted in the country’s total citations which was most evident from 2009 to 2019. During that period, the annual number of citations increased from 883 to 7,403, which coincided with an improvement in the country’s worldwide ranking from 52 to 34 and an increase in the RSI value of total citations from -0.4075 to -0.0016. The RSI value of total citatations was recorded positive in two years only (1996 and 1998), and recently there had been a decline from its best value in 2019 (-0.0016) to its worst in 2022 (-0.5671). The trends in the performance of clinical neuroscience in Saudi Arabia are illustrated in Figure 4 for total citations, Figure 5 for worldwide rankings, and Figure 6 for RSI values.

Comparison to Other Medical Specialties in Saudi Arabia

Total articles and total citations rankings worldwide for 46 medical specialties in Saudi Arabia during 1996-2023 are shown in Table 2.

**Table 2:** Worldwide ranking of 46 medical specialties in Saudi Arabia based on total number of articles and total citations from 1996 to 2023

Worldwide ranking of specialties listed by total articles	Worldwide ranking of total articles	Worldwide ranking of specialties listed by total citations	Worldwide ranking of total citations
Family practice	19	Family practice	22
Health informatics	22	Complementary medicine	26
Complementary medicine	25	Health informatics	28
Transplantation	25	Ophthalmology	28
Ophthalmology	26	Critical care	32
Biochemistry	28	Biochemistry	33
Otolaryngology	29	Transplantation	33
Pharmacology	29	Genetics	34
Genetics	29	Otolaryngology	34
Urology	29	Microbiology	35
Anatomy	30	Pharmacology	35
Medicine (Miscellaneous)	31	Urology	35
Nephrology	31	Anatomy	36
Histology	32	Anesthesiology	36
Dermatology	33	Radiology	36
Hematology	33	Surgery	36
Surgery	33	Haematology	37
Endocrinology	34	Pediatrics	38
Microbiology	34	Pulmonology	38
Health policy	35	Dermatology	39
Infectious disease	35	Emergency medicine	39
Public health	35	Endocrinology	39
Radiology	35	Infectious disease	39
Emergency medicine	36	Internal medicine	39
Pediatrics	36	Medicine (miscellaneous)	39
Pulmonology	36	Neuroscience	39
Oncology	37	Public health	39
Rehabilitation	37	Pathology	40
Anesthesiology	38	Physiology	40
Critical care	38	Geriatrics	41
Cardiology	39	Health policy	41
Immunology	39	Nephrology	41
Internal medicine	39	Oncology	41
Neuroscience	39	Orthopedics	41
Pathology	39	Rehabilitation	41
Embryology	40	Reproductive medicine	41
Orthopedics	41	Histology	42
Epidemiology	42	Cardiology	43
Gastroenterology	42	Embryology	43
Psychiatry	42	Gastroenterology	43
Reproductive medicine	42	Epidemiology	44
Geriatrics and gerontology	43	Hepatology	44
Rheumatology	43	Immunology	44
Hepatology	44	Obstetrics and gynecology	44
Physiology	44	Rheumatology	45
Obstetrics and gynecology	45	Psychiatry	46

**Figure 7:**
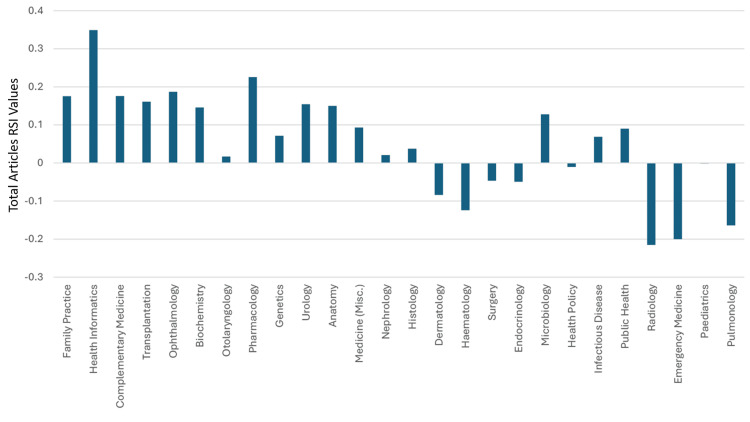
The RSI values of Saudi Arabia’s total articles in the specialties that had a worldwide ranking of 36 or better RSI: Relative Specialization Index; Misc.: miscellaneous This graph has been created by the authors.

**Figure 8:**
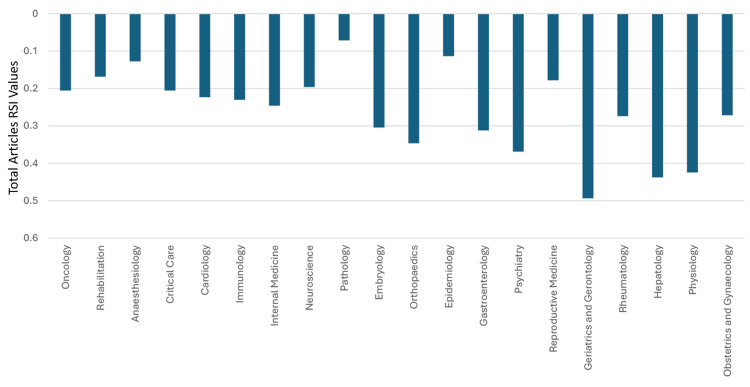
The RSI values of Saudi Arabia’s total articles in specialties that had a worldwide ranking of 37 or worse RSI: Relative Specialization Index This graph has been created by the authors.

The median (range) total articles and total citations for all the specialties were 1,570 (81-53,888) and 24,966 (1293-1008,766), respectively. The median (range) total articles and total citations worldwide rankings were 36 (19-45) and 39 (22-46), respectively. The median (range) RSI values of total articles and total citations were -0.0989 (-0.4933-0.3489) and -0.0976 (-0.5047-0.4755), respectively. Thirty specialties had a worldwide ranking of total articles between 19 and 38, which were better than clinical neuroscience’s worldwide ranking of 39. Hence, based on the total articles worldwide ranking, clinical neuroscience could be considered 31^st^ among the 46 specialties in Saudi Arabia. Twenty-nine of the 46 medical specialties had total articles RSI values that were better than those for clinical neuroscience (>-0.196). Hence, based on the RSI values of total articles, clinical neuroscience could be considered as ranking 30^th^ amongst the 46 specialties in Saudi Arabia. The RSI value for total articles for all the specialties is illustrated in Figures 7-8.

**Figure 9:**
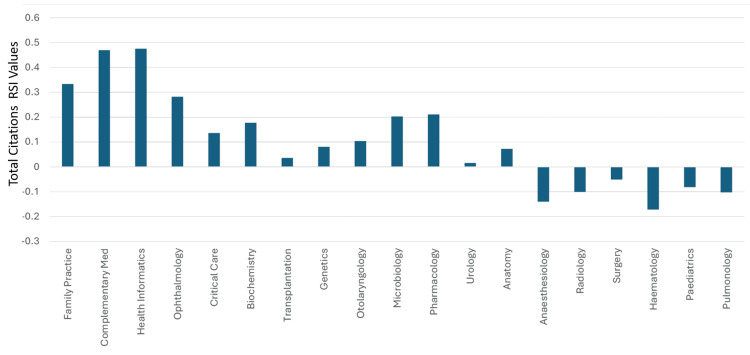
The RSI values of Saudi Arabia’s total citations in the specialties that had a worldwide ranking of 38 or better RSI: Relative Specialization Index This graph has been created by the authors.

**Figure 10:**
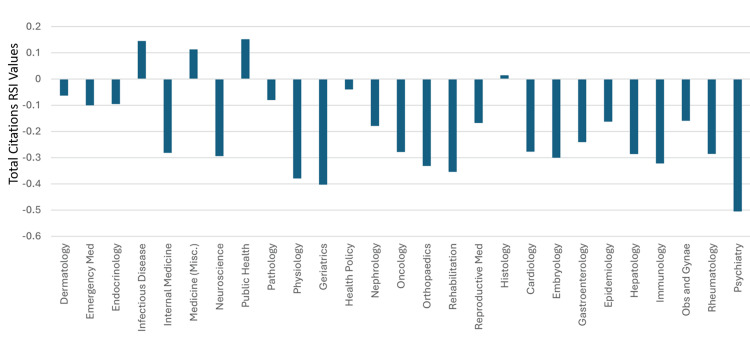
The RSI Values of Saudi Arabia’s total citations in the specialties that had a worldwide ranking of 39 or worse RSI: Relative Specialization Index; Misc.: miscellaneous This graph has been created by the authors.

Nineteen specialties had total citations worldwide rankings between 22 and 38, which was better than clinical neuroscience’s worldwide ranking of 39. Hence, based on total citations worldwide rankings, clinical neuroscience could be considered as ranking 20^th^ among the 46 specialties in Saudi Arabia. Thirty-eight of the 46 medical specialties had total citation RSI values that were better than those for clinical neuroscience (>-0.294). Hence, based on total citation RSI values, clinical neuroscience can be considered as ranking 39^th ^amongst the 46 specialties in Saudi Arabia. The total citations RSI value for 46 specialties in Saudi Arabia during 1996-2023 is illustrated in Figures 9-10.

Comparison to Top 50 Countries in Clinical Neuroscience, Worldwide

The top 50 countries based on total articles and total citations in clinical neuroscience from 1996-2023 are listed in Table 3.

**Table 3:** Worldwide ranking of top 50 countries in clinical neuroscience based on total articles and total citations between 1996 to 2023

Countries ranked by total articles in clinical neuroscience	Worldwide ranking based on total articles	Countries ranked by total citations in clinical neuroscience	Worldwide ranking based on total citations
USA	1	USA	1
Germany	2	UK	2
Japan	3	Germany	3
UK	4	Canada	4
Italy	5	Italy	5
China	6	France	6
Canada	7	Japan	7
France	8	Netherlands	8
Spain	9	Australia	9
Australia	10	Spain	10
Netherlands	11	Sweden	11
India	12	China	12
Switzerland	13	Switzerland	13
South Korea	14	Belgium	14
Brazil	15	Austria	15
Turkey	16	Denmark	16
Sweden	17	South Korea	17
Belgium	18	Brazil	18
Austria	19	Finland	19
Denmark	20	Israel	20
Taiwan	21	Norway	21
Israel	22	Turkey	22
Russia	23	India	23
Poland	24	Taiwan	24
Iran	25	Portugal	25
Finland	26	Poland	26
Norway	27	Greece	27
Czech Republic	28	Ireland	28
Greece	29	New Zealand	29
Portugal	30	Argentina	30
Mexico	31	Hong Kong	31
Argentina	32	Czech Republic	32
Ireland	33	Hungary	33
Hungary	34	Iran	34
Singapore	35	Singapore	35
New Zealand	36	Russia	36
Hong Kong	37	Mexico	37
Egypt	38	South Africa	38
Saudi Arabia	39	Saudi Arabia	39
Chile	40	Chile	40
Thailand	41	Egypt	41
South Africa	42	Thailand	42
Colombia	43	Serbia	43
Romania	44	Colombia	44
Malaysia	45	Malaysia	45
Croatia	46	Romania	46
Serbia	47	Slovenia	47
Slovakia	48	Croatia	48
Nigeria	49	Slovakia	49
Pakistan	50	Luxembourg	50

**Figure 11:**
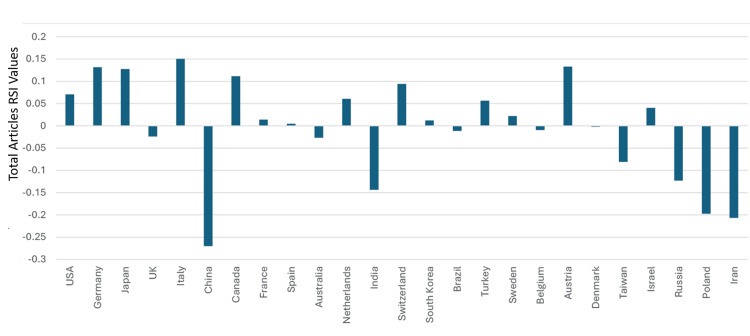
The RSI values of the total articles in the countries that were ranked between one and 25 worldwide in clinical neuroscience specialty RSI: Relative Specialization Index This graph has been created by the authors.

**Figure 12:**
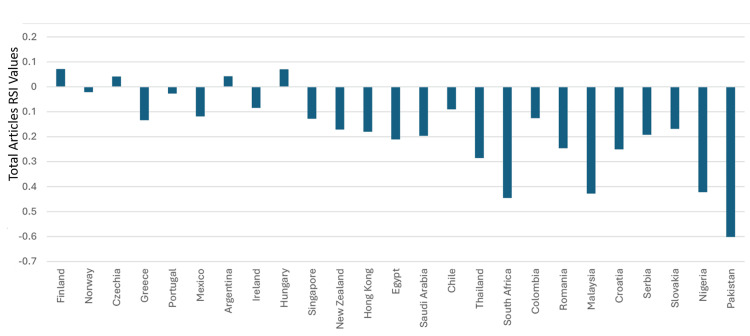
The RSI values of the total articles in the countries that were ranked between 26-50 worldwide in clinical neuroscience specialty RSI: Relative Specialization Index This graph has been created by the authors.

The median (range) total articles and total citations for the top 50 countries in clinical neuroscience were 8,307 (1,200-336,122) and 188,337 (26,016-12517,536), respectively. The median (range) RSI values of total articles and total citations among the top 50 countries in the specialty were -0.054 (-0.6012- 0.1504) and -0.0568 (-0.4464- 1), respectively. Saudi Arabia’s total articles in the specialty were 3,750. The country was ranked 39 and had a total articles RSI value of -0.196. Thirty-eight countries had total articles RSI values that were better than those of Saudi Arabia. Hence, based on total articles RSI values in clinical neuroscience, Saudi Arabia could be considered as ranking 39^th^ in the world. In addition, based on having an RSI≥0.1, clinical neuroscience was considered to have had a strong relative contribution to the total articles in five countries. These were Italy, Austria, Germany, Japan, and Canada. The total articles RSI values for the top 50 countries are illustrated in Figures 11-12.

**Figure 13:**
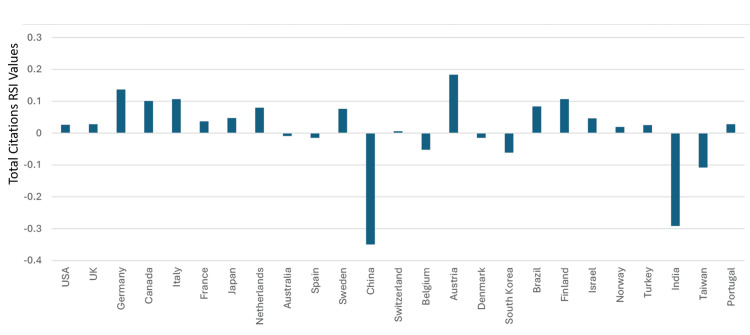
The RSI values of total citations in the countries that were ranked between one and 25 worldwide in clinical neuroscience specialty RSI: Relative Specialization Index This graph has been created by the authors.

**Figure 14:**
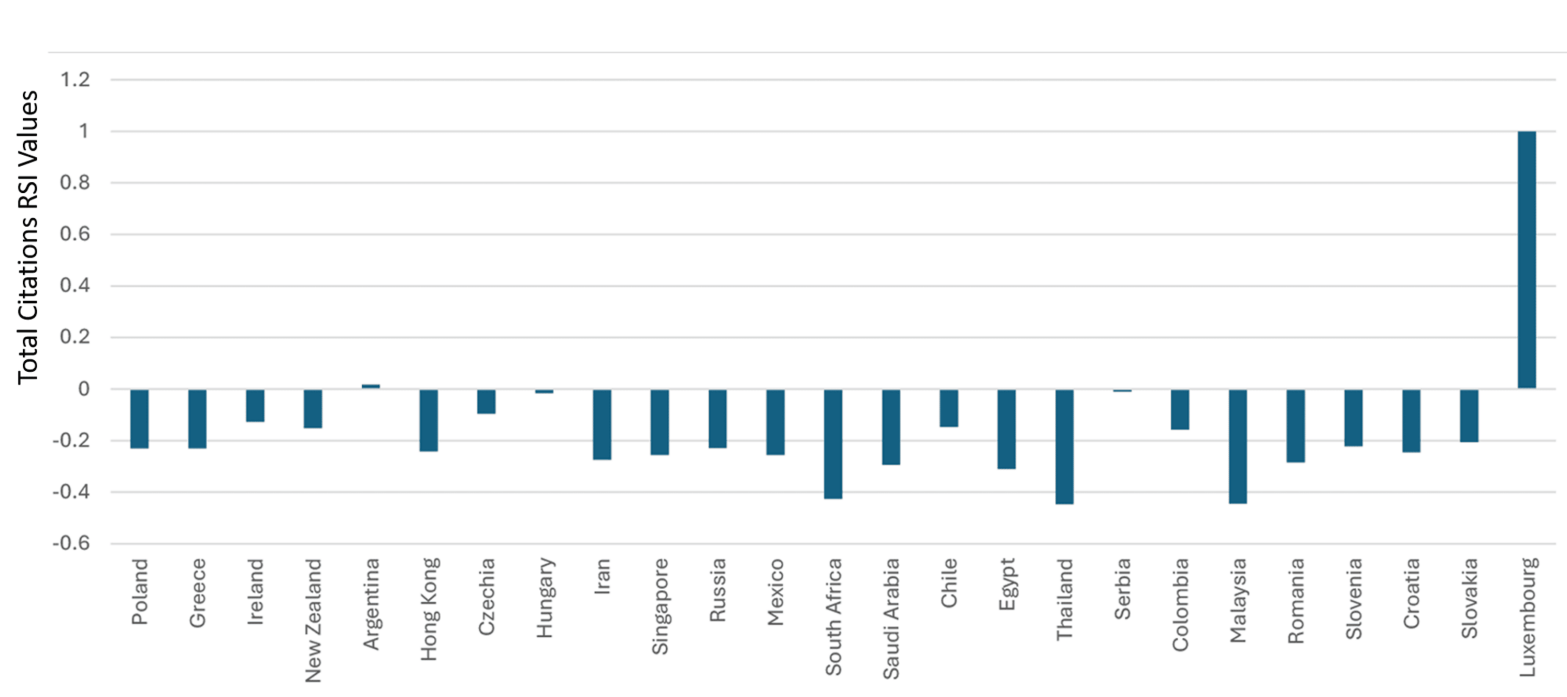
The RSI values of total citations in the countries that were ranked between 26 and 50 worldwide in clinical neuroscience specialty RSI: Relative Specialization Index This graph has been created by the authors.

Saudi Arabia’s total citations in the specialty were 60,903. The country was ranked 39^th^ and had a total citation RSI value of -0.294. Forty-four countries had total citations RSI values that were better than that of Saudi Arabia. Hence, based on total citation RSI values in clinical neuroscience, Saudi Arabia can be considered as ranking 45^th^ in the world. Based on having an RSI≥0.1, clinical neuroscience was considered to have had a strong relative contribution to the total citations in six countries. These were Luxembourg, Austria, Germany, Canada, Italy, and Finland. The total citation RSI values for the top 50 countries are illustrated in Figures 13-14. The correlation analysis between the total articles RSI values and the total articles worldwide rankings for the top 50 countries showed a significant association (R = -0.7136, P < 0.0001). The correlation assessment between the total citation RSI values and total citation worldwide rankings for the top 50 countries also showed a positive link (R = 0.3368) (P = 0.0167).

Discussion

The first medical journal from Saudi Arabia, the Saudi Medical Journal, was established in 1979, and in the years up to 2014, 20 other Saudi journals covering a wide range of medical specialties were launched [2]. Of the latter, only eight journals are currently listed on the SJR website [6]. One of the Saudi journals is a neuroscience journal that is referred to in PubMed as “Neurosciences (Riyadh)” [2, 22]. It is an open-access, peer-reviewed, quarterly publication with nearly 60% of its original articles coming from outside Saudi Arabia [22]. Despite being launched in 1996, the IF of Neurosciences (Riyadh) remains modest, and nearly two-thirds of its research publications are of level evidence (LOE) IV [22].

The RSI measurement in this review allowed us to quantify the share of the clinical neuroscience research out of Saudi Arabia’s total academic productivity. The RSI is a well-recognized bibliometric performance indicator that is calculated using productivity data such as total articles and total citations. Several factors are known to influence a country’s scientific output and would indirectly impact the RSI values. These include many country-specific characteristics such as gross domestic product (GDP) per capita, GDP spending on research and developments (R & D), number of universities among the top 500 in the world, number of Institute of Scientific Information (ISI)-indexed journals, and population size [1, 4]. Furthermore, variation in the amount of publishing between the various medical specialties is well recognized [2,23]. Also well-documented is the wide disparity in productivity among some countries even when the number of specialists is normalized [24].

This review assessed the performance of clinical neuroscience in Saudi Arabia over 28 years (1996-2023). In the last three decades, Saudi Arabia saw considerable growth in the number of researchers, research centers, research resources, and universities [2,4,14,15]. The country also observed an increase in access to collaborative research and training opportunities abroad, as well as the launch of several local journals [2,4,14,22]. The transformation in the country’s research facilities would have most likely affected productivity and impacted the data in the review. We were able to demonstrate that despite a clear-cut increase in the total articles and total citations of clinical neuroscience in Saudi Arabia during 1996-2023, the specialty’s share of the country’s productivity fluctuated and remained negative during most of the period, reflecting a lower than the world’s average. The brief periods of RSI positivity for total articles (2003-2006) and total citations (1996-1998) may have signaled personal efforts by an active cohort of researchers at the time.

We were able to rank clinical neuroscience among the other 45 specialties in Saudi Arabia based on the RSI performance. The specialty ranked 31 amongst the 46 specialties based on the total articles worldwide rankings, and the ranking remained almost unchanged (rank 30) based on the value of the total articles (RSI). In addition, the specialty ranked 20 amongst the 46 specialties based on total citations worldwide rankings. However, the ranking dropped to 39 based on the value of the total citations (RSI). These findings confirm that clinical neuroscience’s share of the productivity in Saudi Arabia compared to the world’s average can be considered mid-range for total articles and low-range for total citations when judged against other medical specialties in the country.

This study showed how the application of the RSI values impacted Saudi Arabia’s worldwide ranking in clinical neuroscience. The country ranked 39 in the world based on the total number of articles in the specialty, and the ranking remained unchanged (rank 39) based on the value of the total articles (RSI). In addition, the country ranked 39 in the world based on total number of citations in the specialty. However, the ranking dropped to 45 based on the value of the total citations (RSI). The latter fits with frequently reported observations that Saudi Arabia’s worst worldwide ranking in clinical neuroscience (134 in the world) was based on citations per article [6]. This was attributed to Saudi Arabian researchers publishing frequently low LOE research in low IF journals and local journals [1,4,11,13,25].

In addition, based on having an RSI ≥ 0.1, we were able to identify countries among top 50 worldwide in which clinical neuroscience had a strong relative contribution to their productivity. For total articles, the countries (and their worldwide ranking) in the order of their RSI values were Italy (5^th^), Austria (19^th^), Germany (2^nd^), Japan (3^rd^), and Canada (7^th^). For total citations, the countries (and their worldwide ranking) in the order of their RSI values were Luxembourg (50^th^), Austria (15^th^), Germany (3^rd^), Italy (5^th^), Finland (19^th^), and Canada (4^th^). Despite the inclusion of three countries that were not highly ranked (Austria, Luxembourg, and Finland) among the groups in which clinical neuroscience had a strong relative contribution, a significant link between RSI values and rankings amongst the top 50 countries in the world in clinical neuroscience was observed.

There are several limitations to this study. The study was dependent on the accuracy of the website search engine SJR. It is possible that there were errors, particularly with multi-national publications. Furthermore, there may have been some specialty and topic overlap. The clinical neuroscience data covered a wide range of journals of varying subspecialties, ages, and IF. The impact of the change in the number of researchers involved over the years that would affect productivity was not examined. It can be argued that the two bibliometric indicators used (total articles and citations) may not provide a true reflection of the quality of research, particularly for publications in local journals. The total articles and citations for the medical specialties in Saudi Arabia were widely ranged. Hence, defining a specialty as having a strong relative contribution to productivity in the country based on RSI≥0.1 alone can be disputed. The influence of the presence of a Saudi specialty association or a specialty journal on the scientific productivity of the various medical specialties was not examined. The impact of the country-specific characteristics on the worldwide ranking in clinical neuroscience was not addressed.

## Conclusions

In conclusion, despite an increase in the number of Saudi Arabia’s total articles and total citations in clinical neuroscience over the years, the specialty’s relative share of the total productivity in the country remains lower than the overall for the specialty worldwide. The specialization performance of the specialty was within the mid-to-low range compared to other medical specialties in Saudi Arabia. In addition, the country's worldwide ranking based on total citations in the specialty dropped when the RSI was used. Clinical neuroscience researchers in Saudi Arabia are encouraged to improve the quality and quantity of their research productivity to be one of the leading medical specialties in Saudi Arabia.
